# Three Discipline Collaborative Radiation Therapy (3DCRT) special debate: In the future, at least 20% of NIH funding for radiotherapy research should be allocated to non‐oncologic applications

**DOI:** 10.1002/acm2.12729

**Published:** 2019-10-01

**Authors:** Krisha Howell, Martha Matuszak, Charles A. Maitz, Subarna H. Eisaman, Laura Padilla, Stephen L. Brown, Michael C. Joiner, Michael M. Dominello, Jay Burmeister

**Affiliations:** ^1^ Department of Radiation Oncology Fox Chase Cancer Center Philadelphia PA USA; ^2^ Department of Radiation Oncology University of Michigan Ann Arbor MI USA; ^3^ Veterinary Health Center University of Missouri Columbia MO USA; ^4^ Department of Radiation Oncology University of Pittsburgh Medical Center, Hillman Cancer Center Pittsburgh PA USA; ^5^ Department of Radiation Oncology Virginia Commonwealth University Richmond VA USA; ^6^ Department of Radiation Oncology Henry Ford Health System Detroit MI USA; ^7^ Department of Oncology Wayne State University School of Medicine Detroit MI USA; ^8^ Gershenson Radiation Oncology Center Barbara Ann Karmanos Cancer Institute Detroit MI USA

## 1 THREE DISCIPLINE COLLABORATIVE RADIATION THERAPY (3DCRT) DEBATE SERIES

Radiation oncology is a highly multidisciplinary medical specialty, drawing significantly from three scientific disciplines — medicine, physics, and biology. As a result, discussion of controversies or changes in practice within radiation oncology involves input from all three disciplines. For this reason, significant effort has been expended recently to foster collaborative multidisciplinary research in radiation oncology, with substantial demonstrated benefit.^1^, ^2^ In light of these results, we endeavor here to adopt this “team‐science” approach to the traditional debates featured in this journal. This article is part of a series of special debates entitled “Three Discipline Collaborative Radiation Therapy (3DCRT)” in which each debate team will include a radiation oncologist, medical physicist, and radiobiologist. We hope that this format will not only be engaging for the readership but will also foster further collaboration in the science and clinical practice of radiation oncology.

## 2 INTRODUCTION

Curative intent indications for radiation therapy (RT) exist outside of the standard paradigm of definitive or adjuvant therapy in oncology. Historically, definitive radiation treatments have included a diverse list of conditions such as acne, ankylosing spondylitis, and tinea capitis to name just a few. Initially, unintended consequences garnered little concern, in part because the slow onset of symptoms made them difficult to detect.^3^ Once toxicities from radiation exposure became evident and better understood, however, therapeutic radiation was largely relegated to malignant conditions. Within the field of oncology, the risk of radiation damage was balanced against the potential for controlling the malignancy.^4^ However, there is evidence supporting the therapeutic use of ionizing radiation for the treatment of a range of specific indications. This raises the question of whether we are appropriately investing in research toward the broader application of radiotherapy to medicine. Perhaps some significant portion, for example, ~20%, of our NIH expenditures on radiotherapy research should be directed toward non‐oncologic applications. This is the subject of this month's 3DCRT debate.

Arguing for the proposition will be Drs. Krisha Howell, Martha Matuszak, and Charles Maitz. Dr. Howell is an Assistant Professor and Assistant Residency and Fellowship Program Director at the Department of Radiation Oncology, Fox Chase Cancer Center, where she specializes in the treatment of sarcoma and gynecologic malignancies. Her research focus includes palliation of bone metastases, hypofractionation in sarcoma, and leadership need identification in physicians. Dr. Matuszak is a medical physicist and serves as an Associate Professor, the Director of Advanced Treatment Planning, and the Director of Clinical Physics at the Brighton Center for Specialty Care in the Department of Radiation Oncology at the University of Michigan. Her research focuses on incorporation of functional imaging and other biomarkers into treatment plan optimization. Dr. Matuszak is also highly involved in in‐house and national clinical trials, mostly focusing on lung cancer and response‐based adaptive therapy. Dr. Maitz is a veterinary radiation oncologist, Assistant Professor of Veterinary Medicine and Surgery, and a Research Scientist at the MU Research Reactor at the University of Missouri. His research focuses on translational high LET therapy and radiopharmaceutical dosimetry.

Arguing against the proposition will be Drs. Subarna Eisaman, Laura Padilla, and Stephen Brown. Dr. Eisaman is the clinical director and assistant professor with the University of Pittsburgh Medical Center (UPMC) Hillman Cancer Center Department of Radiation Oncology at the J. Murtha Pavilion in Johnstown, PA. She serves as co‐chair of the Radiation Oncology Lung and Lymphoma Via Oncology Pathways Physician Advisory Committee. Her clinical practice includes treatment of breast, GYN, lung, CNS, head and neck, skin, and musculoskeletal malignancies. Dr. Padilla is a medical physicist in the Department of Radiation Oncology at Virginia Commonwealth University. She has an Assistant Professor appointment and is the Associate Program Director of the Medical Physics graduate program. Her research focuses on uses of surface imaging in radiation oncology, workflow and process improvements, and new educational strategies in medical physics. Dr. Brown is a senior scientist in the Department of Radiation Oncology at Henry Ford Health System, co‐leader of the Translational Oncology Group at the Henry Ford Cancer Institute, and Professor of Oncology at Wayne State University School of Medicine. He studies physiological changes after radiation and explores strategies to exploit differences between tumor and normal tissue responses to improve therapeutic gain.

## 3 OPENING STATEMENTS

### 3.A Krisha Howell, MD; Martha Matuszak, PhD; Charles Maitz, DVM, PhD

Advances in scientific understanding and treatment of cancer have led to improved patient outcomes and quality of life.^5^ Despite the current status, a continued pledge to research funding and innovation is needed within all healthcare. The primary federal agency charged with conducting and supporting biomedical and behavioral research is the National Institutes of Health (NIH). For the fiscal year 2019, the NIH has estimated a program level total of $39.3 billion.^6^ Notably, however, a 2013 analysis (the most recent analysis with expenditures broken down specific to Radiation Oncology principle investigators) suggests that <0.3% of NIH‐funded principal investigators work in the field of radiation oncology and secure a limited portion of the funding provided for cancer research by the NIH.^7^

In the third annual *State of Cancer Care in America* report, the American Society of Clinical Oncology (ASCO) describes challenges and opportunities facing the U.S. cancer care system. Exciting progress in treatment is set against the backdrop of increasingly unsustainable costs and volatile practice environments.^5^ Furthermore, the aging population transforms an increasing number of patients whose cancer will be complicated by chronic diseases. The ASCO findings may be extrapolated to overarching healthcare in the United States and to the patient population suffering from non‐neoplastic conditions and benign tumors. In the anticipated future of NIH funding and healthcare in the United States, there is increasing impetus to attain sustainable care: reducing inefficiencies and improving outcomes. In light of limited resources and increasing demand, this is a challenging task. We must find better ways of allocating the resources we have, and focusing on what can make the greatest impact to patients.^8^

The majority of patients treated with external beam RT are treated for cancer; however, the same treatment infrastructure can be used to administer RT to patients with a variety of non‐neoplastic conditions and benign tumors. Indications for RT of benign disease have been identified as: acute/chronic inflammatory disorders, acute/chronic painful degenerative diseases, hypertrophic (hyperproliferative) disorders of soft tissues, functional diseases, among other indications.^9^ Preclinical evidence even indicates that some anticancer radiotherapy techniques can be effective in treating infectious diseases.^10^

RT is a well‐accepted and frequently practiced treatment for several benign diseases in Germany.^11^ Outside of Europe, however, the use of RT to treat benign disease is often regarded with skepticism. Only about ten of a potentially 100 indications for RT of benign diseases would be treated by more than 90% of North American radiation oncologists, according to a 1990 survey.^9^ Few benign treatment indications are generally accepted, defined as yielding a positive approval of over 50% worldwide. Some examples include postoperative prophylaxis of keloids and heterotopic ossification (HO) and treatment of Graves' orbitopathy. Within the United States, trigeminal neuralgia, arteriovenous malformation, acoustic neuroma, and meningioma are customarily accepted for fractionated external beam RT or stereotactic radiosurgery. Other indications, in contrast, reveal a divergent acceptance, for example, RT of painful osteoarthrosis (Eastern Europe, 85% vs United States, 5%).^11^ Beyond those widely and regionally accepted indications, there exist a subset of nontraditionally explored indications (movement disorders, rhizotomy outside of the brain, psychiatric disorders, and cardiac arrhythmias) that have been favorably reported in small patient cohorts.^12^, ^13^, ^14^

Some estimates predict that upward of one‐third of all patients undergoing total hip arthroplasty will develop HO, or approximately 50 000–60 000 patients in the United States alone.^11^ RT was first used in 1981 in patients at high risk of HO. Several randomized and some prospective randomized trials support RT as a prophylactic treatment of HO and support a dose de‐escalation to 7 Gy in a single fraction.^15^, ^16^, ^17^, ^18^, ^19^, ^20^, ^21^, ^22^, ^23^ It is possible that RT could provide a useful treatment modality with low acute toxicity for patients with benign conditions in an age group where the risk of late‐term toxicity is not clinically relevant.^4^ Randomized trials of prophylactic therapy for this condition demonstrated that both RT and NSAIDs produced very low rates of HO. In a meta‐analysis, RT reduced the risk of Brooker grades 3–4 HO significantly better than NSAIDs (0.9% vs 2.9%, *P* = 0.043). For overall HO, there was no significant difference in outcome between the two measures.^16^

Current radiobiological evidence suggests RT at the low to intermediate doses used for many benign conditions will cause cell and molecular changes, although these will be largely asymptomatic during the therapy and for the acute time period thereafter. Doses used for treating benign tumors are much closer to the standard cancer therapeutic range, and for some indications, for example, trigeminal neuralgia, the dose is very high (70–90 Gy) though delivered to a very small volume. Hence, since the total integral dose of radiation is significantly less than that delivered to most patients treated for malignant tumors, the chance of overt effects related to dose and radiation quality is low.^4^ In the end, given the age range of most patients and the relatively low RT doses and/or fields employed for benign conditions, the risks of RT may be lower than the risks of alternative pertinent therapies such as anti‐inflammatory drugs and other interventions.^4^

It is likely that most of the contention against RT for benign conditions, and the decline in its service, is the fear of the risk of radiation‐induced cancer (RIC).^4^ Over the past several decades, well‐conducted epidemiological studies and large patterns of care studies have been performed including studies of Japanese atomic bomb survivors who were exposed to whole‐body irradiation. Risk of RIC appeared to increase approximately linearly with dose. The risk was also proportional to the radiation field size and significantly reduced as the age at initial radiation exposure increased.^24^ Genetic data now available also support potential germline mutations that may exist in cancer survivors predisposing this population to a greater likelihood of secondary cancers than that of the general public.^24^

Evidence of RT for many benign conditions is comprised mainly of case reports or small single institution retrospective series. The radiobiological mechanisms to explain the success in controlling the varied indications are likely a rather complex collaboration of several effects.^9^ Recent research in radiotherapy of cancer has resulted in a much greater understanding of the effects of radiation on the immune system. These immune effects could have significant impacts on the role of RT in the treatment of non‐neoplastic, or benign diseases, as well. More basic research has to be initiated or strengthened, and controlled clinical multicenter studies conducted to confirm basic research data, and prove treatment efficacy. Current radiation prescriptions vary with regard not only to the single and total doses but also to fractionation schedules and treatment techniques. The last written recommendations for the treatment of nonmalignant disease in the United States were made by the Bureau of Radiologic Health in 1977.^4^ Thus, no treatment standard has been established in many of the indications.^11^

It is time for those working in Radiation Oncology departments and investigating different funding opportunities to embrace non‐oncologic applications that can be served by the great technological advances in our field. Acceptance of stereotactic radiosurgery and stereotactic body RT for various benign conditions including ventricular tachycardia are evidence of the increasing acceptance that more advanced radiation delivery can minimize dose to normal tissues and provide noninvasive treatments to benign conditions for which surgical and other treatments are fraught with increased complications and/or costs. The use of radiation for these expanded indications brings in new collaborators, commercial and social interest, and drives new technological advancements that can then be applied to oncologic and non‐oncologic applications alike. Therefore, based on the above facts, future funding of radiotherapy by the NIH should allocate at least 20% of its funds to non‐oncologic applications.

### 3.B Subarna Eisaman, MD, PhD; Laura Padilla, PhD; Stephen Brown, PhD

There are many reasons we disagree that “in the future, at least 20% of NIH funding for radiotherapy research should be allocated to non‐oncologic applications.” First and foremost, research dollars should be dispersed based on merit. Although there are several valuable non‐oncologic applications of radiotherapy (treatments for trigeminal neuralgia, keloids, arteriovenous malformations, etc.), and more are sure to arise, these should not have a pre‐allocated portion of the radiotherapy research funds. NIH has historically supported research based on scientific review using well‐publicized criteria and metrics: Significance, Innovation, Approach, Investigators, and Environment. Then, at the council level, selection follows programmatic priorities. This strategy promotes sound scientific research and its value should not be disregarded; there is no need for a shift in paradigm. It is important to highlight that in times of national need, NIH dollars for radiation research studies focused on non‐oncologic areas are made available. This was the case after the horrific 9–11 terrorist attacks, when there was an urgent call for funding of research for radiation injury countermeasures. However, of note, these are new dollars and are thus not in competition with oncology‐focused research. As cancer remains the second leading cause of death in the United States, with 595 919 cancer deaths reported in 2015, radiotherapy research for oncologic uses is still very much needed.

In fact, using the 2018 estimates of NIH funding distribution among various Research, Conditions, and Disease Categories (RCDC), 80% of all NIH RCDC funds were already utilized for non‐oncologic research including $5749M on brain disorders, $643M on cardiovascular disease, $466M on depression, $627M on kidney disease, $4935M on rare diseases, and $13 720M on general clinical research.^25^ Only 20% ($41 420M of the $205 812M) of the total RCDC funds was used toward cancer, and of those, only 0.8% ($337M) was allocated to RT funding.^25^ Radiotherapy plays a critical role in the management of nearly two‐thirds of all cancers. In many cases, it is the definitive, curative treatment modality, providing an alternative to surgery. Hence, the allocated NIH funding is already disproportionately low given the clinical relevance of our field; there is no rationale for further decreasing the radiation oncology funding by allocating a fixed 20% for non‐oncologic applications.

Furthermore, NIH‐funded projects in radiation oncology such as those leading to the development of 3D conformal radiotherapy have paved the way for our current clinical oncology practice. The application of 3D conformal radiotherapy signified a major improvement over conventional 2D RT. Using more conformal techniques for dose distribution, the radiation beams are optimized to deliver a higher dose to specified target volumes, while reducing the dose to adjacent organs at risk (OARs). The NSABP Protocol R‐03 trial studying pre‐ and postoperative chemoradiation for rectal cancer used traditional four‐field box 2D RT in 1997 and reported grade 3 or higher diarrhea (39% preoperative arm) as their principal toxicity.^26^ With 3D‐CRT, grade 3 or higher diarrhea was down to 6.3% for 859 similar rectal cancer cases.^27^ It is clear, from these data and others, that better physical targeting and conformality of radiotherapy treatments can improve patient outcomes. Beyond 3D conformal radiotherapy, the evolution of modern radiation oncology has continued with the advent of image‐guided RT (IGRT), intensity‐modulated RT (IMRT), volumetric‐modulated arc therapy (VMAT), linear accelerators with MR capabilities, etc. These technologies have radically improved how accurately and precisely we can target and treat a given anatomical volume. It stands to reason that this may lead some to believe that the technology is reaching a plateau, and funding could be better used elsewhere. However, one must look deeper than just anatomy and equipment capabilities and into biology. Precision medicine is based on precise delivery at the molecular level, and we still have a long way to go to truly understand the mechanisms and interactions, and how to best use them to our advantage.

Although the mechanisms may not be fully understood, we do know that radiotherapy has the ability to alter the predominant method of cell kill with anatomic precision through proper targeting and adaptive dose fractionation schemes. This flexibility makes it an invaluable tool to optimize the therapeutic ratio within an individual tumor by modifying the local tumor microenvironment and the systemic immune response. Allowing the radiobiology to inform the treatment design could augment the therapy's efficacy by including molecular targeted therapy, or complementing the treatment with adjuvant therapy for tumors that are identified to be genomically predisposed to radioresistance. Both molecular and immunologic targeted agents can be used to sensitize tumor cells to radiotherapy. For example, EGFR‐inhibitors like erlotinib, and PARP‐inhibitors like inipirib, can target radioresistant tumor cells to enhance the effect of radiotherapy. Immunotherapy targeted agents such as PD‐1, and PD‐L1 targeted drugs like durvalumab and nivolumab, boost immune T‐cell response and may promote abscopal effects of radiotherapy. This could transform radiotherapy from being exclusively a localized treatment into a more systemic one through the induction of treatment effects in distant metastatic sites outside of the radiation field. These potentially revolutionary cancer treatments require further investigation, and their funding could be compromised by allocating NIH money away from oncologic radiotherapy research.

Through the information presented in this statement, it is clear that more radiotherapy research and clinical trials are imperative for the benefit of future cancer patients. Although there are many valuable non‐oncological applications of radiotherapy, their research should be funded based on merit, not by pre‐allocating money away from radiation oncology. The field of radiation oncology, in its multidisciplinary and synergistic nature, needs suitable NIH financial support to properly address one of the leading causes of death in the country. Moreover, while it is important to ensure that radiotherapy uses are expanded beyond radiation oncology to diversify the field and secure its future, we should not propose this to be done at the potential detriment of patient care and scientific quality.

## 4 REBUTTAL

### 4.A Krisha Howell, MD; Martha Matuszak, PhD; Charles Maitz, DVM, PhD

We appreciate our colleagues' thoughtful position against allocating at least 20% of NIH funding to radiotherapy into non‐oncologic applications however, we respectfully disagree with their position to accept the status quo as sufficient. Having said that, we do whole‐heartedly agree with their position that research dollars should be dispersed based on merit. The intense scrutiny of grant proposals and distribution of funds developed by the NIH, while it may have some inherent issues, is a robust vetting process to determine the best projects and investigators most likely to succeed. In addition to rewarding grants based on these merits, however, the NIH is fully capable of emphasizing a particular disease site or concept. Providing such financial incentives will help further guide or attract those with merit to the demarcated disease or condition of need. Furthermore, the concern posed by our colleagues that a pre‐allocated portion of radiotherapy funds be directed to non‐oncology diseases, while well intended, is an optimistic vision of our guaranteed funding and a myopic one of the future prospects of our field. First off, the NIH explicitly states that it “does not expressly budget by category. The annual estimates reflect amounts that change as a result of science, actual research projects funded…(t)he research categories are not mutually exclusive. (And) I(i)ndividual research projects can be included in multiple categories.”^25^ As stated in our opening paragraph, there is historically low funding for grants in Radiation Oncology. The analysis by Steinberg et al. identified 197 grants for which the principle investigator was affiliated with Radiation Oncology. In 79% of the grants, the research topic fell into the field of Biology, 13% in Medical Physics, and only 7.6% of the proposals were clinical investigations.^7^ The lack of physician scientists with active grants in the discipline of Radiation Oncology raises concerns for the advancement and translation of the basic science into clinical practices. Collaboration among other fields and other diseases could be a fruitful partnership in securing more funding and forwarding RT as a science. The advancements in RT for non‐oncologic applications will undoubtedly circle back to benefit the oncologic patient as well.

Our colleagues opined that in the study of RT for non‐oncology indications, funding should only be directly increased if a catastrophic event (another 9–11 urgent emergency) occurs or an extreme national need is felt. We would argue that, first of all, if investment is spurred only by a catastrophic event, then we have missed an opportunity to provide appropriate care of our patients. To help put this argument in context, the World Health Organization (WHO) has stated that antibiotic resistance is one of the biggest impending threats we are facing today in global health.^28^ There is some evidence that RT may be able to treat some resistant bacterial, fungal, and viral (including HIV) infections.^10^, ^29^ However, there has not yet been a concerted push to fund this application of RT. Second, is not the magnitude of patients suffering from these aforementioned conditions already a concern? At what point do we become alarmed that a modality is not being further explored that could control their condition(s)? And third, is not the state of the American healthcare system at a point of crisis in the here and now? If there is a possibility that the treatment of a condition or episode may be better managed by RT, as some data have shown in cancer diagnosis compared to surgery and/or targeted agents, should it not be explored as a definitive and cost‐effective measure in other, relevant diseases?^30^

Our colleagues readily point out that NIH‐funded projects in Radiation Oncology have historically paved the way for improved technological advances. We agree with this sentiment and are optimistic that our advancements may be reapplied to the non‐oncology disciplines approximately a century after these disciplines largely abandoned it out of concerns for toxicity. This time we can apply RT with greater accuracy and knowledge to ablate a dysfunctional electrical pathway in the heart or minimize radiation side effects in a young patient with recurrent keloids to name just two examples. We are aware, however, that specific caution still needs to be exercised in young patients, and that children should only be treated in emergency situations where no other therapeutic solutions seem possible.^24^

In closing our rebuttal, we also conclude along with our colleagues that precision medicine is based on an improved understanding of the mechanisms and interactions at the cellular level. What we disagree upon, however, is that this understanding can only come from remaining affixed to the notion of siloed advancement of radiotherapy by Radiation Oncologists in oncology alone. At the time of this writing, the American Academy of Neurology Annual meeting in Philadelphia had just concluded. One of the notable findings at this meeting was that female multiple sclerosis patients have a reduced risk of relapse in the postpartum period if having breastfed their child. While the decline in multiple sclerosis severity surrounding pregnancy was expected, the drop from breast‐feeding is not as easily explained nor inherently expected in this autoimmune disease. While multiple sclerosis and pregnant patients may be very remote from RT at this moment in time, the mechanism of the immune response is of interest to and heavily studied in our field. We propose that expanding our collaborative partners, broadening our area of interest, and disrupting our notion that the study of radiotherapy must stay in the “four‐field” box of oncology will enable us to embrace the larger objective of healing the patient as a whole.

### 4.B Subarna Eisaman, MD, PhD; Laura Padilla, PhD; Stephen Brown, PhD

Our colleagues eloquently document the need for research dollars in non‐oncologic uses, but fail to provide reasons to support their stated view that a percentage (~20%) of scarce NIH funds currently allocated for radiotherapy should be diverted to non‐oncologic research. We agree that non‐oncologic research is important. In fact, we provide some of the same arguments as our colleagues in support of the need for further studies to improve currently accepted non‐oncologic uses of radiotherapy, as well as investigate less explored applications, such as radiotherapy for psychiatric disorders. We wholeheartedly agree that “The use of radiation for these expanded indications brings in new collaborators, commercial and social interest, and drives new technological advancements” — it would diversify and expand the scope of our field and be beneficial to all of those involved. However, this transition needs to be done on the shoulders of quality research; proposals should be funded, regardless of whether they are for oncologic or non‐oncologic applications of radiation, based on their excellence in “Significance, Innovation, Approach, Investigators, and Environment” when compared to the rest. Perhaps if the argument is that quality research is not being funded for non‐oncologic applications of radiation, the discussion should be shifted toward how proposals dealing with medical uses of radiation are evaluated, not how much money should be pre‐allocated away from one application to the other. There remains a lot of work and innovation to be done in radiation oncology that could improve patient outcomes and at the same time provide valuable information for non‐oncologic applications, or as our colleagues said” …that can then be applied to oncologic and non‐oncologic applications alike.”

As the group arguing for the proposition also pointed out, “Recent research in radiotherapy of cancer has resulted in a much greater understanding of the effects of radiation on the immune system. These immune effects could have significant impacts on the role of RT in the treatment of non‐neoplastic or benign diseases, as well.” As this statement alludes, and has been noted in the literature^31^ and throughout this debate before, cancer research can provide valuable information for other applications of radiotherapy. As cancer is one of the leading causes of death at a national and international level, well‐designed, strong projects investigating how to achieve the greatest therapeutic power with minimal side effects can have great patient impact and their funding should not be jeopardized by prestipulated allocations. However, since we all agree that the field has the potential to affect the lives of many patients beyond cancer, radiation oncology proposals pursuing funding should include budgeted tissue collections for genomic studies and other means of contributing to big data resources available to the scientific community. This could help build centralized databases to inform precision oncology and genomic guided radiotherapy studies,^32^ as well as contain identifiable patient traits that might aid the design of non‐oncologic radiotherapy courses and predict outcomes as studies for new applications arise.

We also concur with our colleagues that the use of ionizing radiation poses risks, some of which are not completely understood. Consequently, research dollars are needed. Once again, our contention, not refuted by our opposition, is that the allotment of funds for such research needs to be weighed against other priorities according to the NIH guidelines of peer review.

Overall, we agree there are many worthwhile non‐oncologic radiation research venues that may merit funding. The allocation of NIH‐funds should continue to be based on scientific merit. In the current environment of limited NIH funding, there is no reason to allot at least 20% of the radiation research dollars from already underfunded oncologic radiation research to non‐oncologic ends.

## CONFLICT OF INTEREST

The authors declare no conflicts of interest.

## References

[1] Burmeister J , Tracey M , Kacin S , Dominello M , Joiner M . …Of radiation oncology, biology, and physics. Int J Radiat Oncol Biol. 2018;100:1289–1290. 10.1016/j.ijrobp.2018.01.045 PMC5937706 29722663

[2] Burmeister J , Tracey M , Kacin S , Dominello M , Joiner M . Improving research in radiation oncology through interdisciplinary collaboration. Rad Res. 2018;190:1–3. 10.1667/RR15023.1 PMC6052437 29693501

[3] Reed A . The history of radiation use in medicine. J Vasc Surg. 2011;53(1):3S–5S. 20869835 10.1016/j.jvs.2010.07.024

[4] Taylor R , Hatfield P , McKeown S , Prestwich R , Shaffer, R. A review of the use of radiotherapy in the UK for the treatment of benign clinical conditions and benign tumours. London, UK: The Royal College of Radiologists 2015. ISBN:978‐1‐905034‐66‐6.

[5] Kirkwood MK . The state of cancer care in America, 2016: a report by the American Society of Clinical Oncology. J Oncol Pract. 2016;12(4):339–383. 26979926 10.1200/JOP.2015.010462 PMC5015451

[6] Johnson JA , Sekar K. National Institutes of Health (NIH) Funding: FY1994‐FY2020. Congressional Research Service. Washington, DC: Congressional Research Service; 2019.

[7] Steinberg M , McBride WH , Vlashi E , et al. NIH funding in radiation oncology – A snapshot. Int J Radiat Oncol Biol Phys. 2013;86(2):234–240. 23523324 10.1016/j.ijrobp.2013.01.030 PMC3646925

[8] Wait S , Han D , Muthu V , et al. Towards sustainable cancer care: Reducing inefficiencies, improving outcomes – A policy report from the All.Can initiative. J Canc Policy. 2017;13:47–64.

[9] Micke O , Seengenschmiedt MH ; German Working Group on Radiotherapy in Germany . Consensus guidelines for radiation therapy of benign diseases: a multicenter approach in Germany. Int J Radiat Oncol Biol Phys. 2002;52(2):496–513. 11872298 10.1016/s0360-3016(01)01814-4

[10] Dadachova E , Casadevall A . Radiolabeled antibodies for therapy of infectious diseases. Microbiol Spectr. 2014;2(6):0023. 25599011 10.1128/microbiolspec.AID-0023-2014 PMC4294273

[11] Seegenschmiedt MH , Katalinic A , Makoski H , et al. Radiation therapy for benign diseases: Patterns of care study in Germany. Int J Radiat Oncol Biol Phys. 2000;47(1):195–202. 10758324 10.1016/s0360-3016(99)00537-4

[12] Li G , Patil C , Adler JR , et al. CyberKnife rhizotomy for facetogenic back pain: a pilot study. Neurosurg Focus. 2007;23(6):E2. 10.3171/FOC-07/12/E2 18081475

[13] Park S , Lee JK , Kim C , et al. Gamma‐knife subcaudate tractotomy for treatment‐resistant depression and target characteristics: a case report and review. Acta Neurochir. 2017;159:113–120. 27900544 10.1007/s00701-016-3001-3

[14] Cuculich PS , Schill MR , Kashani R , et al. Noninvasive cardiac radiation for ablation of ventricular tachycardia. N Engl J Med. 2017;377:2325–2336. 29236642 10.1056/NEJMoa1613773 PMC5764179

[15] Burd TA , Hughes MS , Anglen JO . Heterotopic ossification prophylaxis with indomethacin increases the risk of long‐bone non‐union. J Bone Joint Surg Br. 2003;85(5):700–705. 12892193

[16] Kienapfel H , Koller M , Wüst A , et al. Prevention of heterotopic bone formation after total hip arthroplasty: a prospective randomised study comparing postoperative radiation therapy with indomethacin medication. Arch Orthop Trauma Surg. 1999;119(5–6):296–302. 10447627 10.1007/s004020050414

[17] Sell S , Willms R , Jany R , et al. The suppression of heterotopic ossifications: radiation versus NSAID therapy – a prospective study. J Arthroplasty. 1998;13(8):854–859. 9880175 10.1016/s0883-5403(98)90189-9

[18] Kölbl O , Knelles D , Barthel T , Kraus U , Flentje M , Eulert J . Randomized trial comparing early postoperative irradiation vs. the use of nonsteroidal anti‐inflammatory drugs for prevention of heterotopic ossification following prosthetic total hip replacement. Int J Radiat Oncol Biol Phys. 1997;39(5):961–966. 9392532 10.1016/s0360-3016(97)00496-3

[19] Kölbl O , Knelles D , Barthel T , Raunecker F , Flentje M , Eulert J . Preoperative irradiation versus the use of nonsteroidal anti‐inflammatory drugs for prevention of heterotopic ossification following total hip replacement: The results of a randomized trial. Int J Radiat Oncol Biol Phys. 1998;42(2):397–401. 9788422 10.1016/s0360-3016(98)00204-1

[20] Moore KD , Goss K , Anglen JO . Indomethacin versus radiation therapy for prophylaxis against heterotopic ossification in acetabular fractures: a randomised prospective study. J Bone Joint Surg Br. 1998;80(2):259–263. 9546456 10.1302/0301-620x.80b2.8157

[21] Bremen‐Kuhne R , Stock D , Franke C . Indomethacin – short term therapy vs. single low dosage radiation for prevention of periarticular ossifications after total hip endoprosthesis. Z Orthop Ihre Grenzgeb. 1997;135(5):422–429. 9446435 10.1055/s-2008-1039411

[22] Knelles D , Barthel T , Karrer A , Kraus U , Eulert J , Kölbl O . Prevention of heterotopic ossification after total hip replacement. A prospective, randomised study using acetylsalicylic acid, indomethacin and fractional or single‐dose irradiation. J Bone Joint Surg Br. 1997;79(4):596–602. 9250745 10.1302/0301-620x.79b4.6829

[23] Pakos E , Ioannidis J . Radiotherapy vs. nonsteroidal anti‐inflammatory drugs for the prevention of heterotopic ossification after major hip procedures: a meta‐analysis of randomized trials. Int J Radiation Oncology Biol Phys. 2004;60(3):888–895. 10.1016/j.ijrobp.2003.11.015 15465207

[24] Leer JW , van Houtte P , Seegenschmiedt H . Radiotherapy of non‐malignant disorders: Where do we stand? Radiother Oncol. 2007;83:175–177. 17490769 10.1016/j.radonc.2007.04.008

[25] https://report.nih.gov/categorical_spending.aspx. Accessed January 30, 2019.

[26] Hyams DM , Mamounas EP , Petrelli N , et al. A clinical trial to evaluate the worth of preoperative multimodality therapy in patients with operable carcinoma of the rectum: a progress report of National Surgical Adjuvant Breast and Bowel Project Protocol R‐03. Dis Colon Rectum. 1997;40:131–139. 9075745 10.1007/BF02054976

[27] Wee CW , Kang HC , Wu HG , et al. Intensity‐modulated radiotherapy versus three‐dimensional conformal radiotherapy in rectal cancer treated with neoadjuvant concurrent chemoradiation: a meta‐analysis and pooled‐analysis of acute toxicity. Jpn J Clin Oncol. 2018;48(5):458–466. 29554287 10.1093/jjco/hyy029

[28] World Health Organization . Global action plan on antimicrobial resistance. Geneva, Switzerland: WHO Document Production Services; 2015.

[29] Helal M , Dadachova E . Radioimmunotherapy as a novel approach in HIV, bacterial, and fungal infectious diseases. Cancer Biother Radiopharm. 2018;33:330–335. 30133305 10.1089/cbr.2018.2481 PMC6207155

[30] Lester‐Coll N , Rutter CE , Bledsoe TJ , Goldberg SB , Decker RH , Yu JB . Cost‐effectiveness of surgery, stereotactic body radiation therapy, and systemic therapy for pulmonary oligometastases. Int J Rad Onc Biol Phys. 2016;95(2):663–672. 10.1016/j.ijrobp.2016.01.020 27055395

[31] Coleman C , Prasanna P , Bernhard E , et al. Accurate, precision radiation medicine: a meta‐strategy for impacting cancer care, global health, and nuclear policy and mitigating radiation injury from necessary medical use, space exploration, and potential terrorism. Int J Radiation Oncol Biol Phys. 2018;101(2):250–253. 10.1016/j.ijrobp.2018.02.001 29726348

[32] Hall WA , Bergom C , Thompson RF , et al. Precision oncology and genomically guided radiation therapy: a report from the American Society for Radiation Oncology/American Association of Physicists in Medicine/National Cancer Institute Precision Medicine Conference. Int J Radiation Oncol Biol Phys. 2018;101(2):274–284. 10.1016/j.ijrobp.2017.05.044 28964588

